# Cold shock CspA and CspB protein production during periodic temperature cycling in *Escherichia coli*

**DOI:** 10.1186/1756-0500-6-248

**Published:** 2013-07-02

**Authors:** Tina Ivancic, Polona Jamnik, David Stopar

**Affiliations:** 1 Laboratory of Microbiology, Department of Food Science and Technology, Biotechnical Faculty, University of Ljubljana, Večna pot 111, 1000 Ljubljana, Slovenia; 2 Laboratory of Biotechnology, Microbiology and Food Safety, Department of Food Science and Technology, Biotechnical Faculty, University of Ljubljana, Jamnikarjeva 101, Ljubljana, 1000, Slovenia

**Keywords:** CspA-FLAG, CspB-FLAG, Temperature cycling, Protein degradation, *cspA* and *cspB* transcription

## Abstract

**Background:**

Temperature is an important environmental factor which can dramatically affect biochemical processes in bacteria. Temperatures above optimal cause heat shock, while low temperatures induce cold shock. Since the physiological response of the bacterium *Escherichia coli* to slow temperature fluctuation is not well known, we investigated the effect of periodic temperature cycling between 37° and 8°C with a period of 2 h on proteome profile, cold shock CspA and CspB protein and gene production.

**Results:**

Several proteins (i.e. succinyl-CoA synthetase subunit alpha, periplasmic oligopeptide-binding protein, maltose-binding periplasmic protein, outer membrane porin protein, flavodoxin-1, phosphoserine aminotransferase) were up or down regulated during temperature cycling, in addition to CspA and CspB production. The results indicate that transcription of *cspA* and *cspB* increased during each temperature downshift and consistently decreased after each temperature upshift. In sharp contrast CspA-FLAG and CspB-FLAG protein concentrations in the cell increased during the first temperature down-shift and remained unresponsive to further temperature fluctuations. The proteins CspA-FLAG and CspB-FLAG were not significantly degraded during the temperature cycling.

**Conclusion:**

The study demonstrated that slow periodic temperature cycling affected protein production compared to cells constantly incubated at 37°C or during classical cold shock. Bacterial *cspA* and *cspB* mRNA transcript levels fluctuated in synchrony with the temperature fluctuations. There was no corresponding pattern of CspA and CspB protein production during temperature cycling.

## Background

Most of our knowledge about the cold shock response of bacteria is obtained from experiments in which cells are fast cold shocked and left at low temperatures until they recover. In the environment, however, cells are exposed to changes in temperature on a regular diurnal or seasonal basis, as well as on less predictable time scales. Temperature fluctuations can dramatically affect the physiology of bacterial cultures [1-7]. Under a fluctuating temperature regime, metabolic efficiency may decrease [8], the abundance of *Escherichia coli* may differ [9] and the survival of *E. coli* O157:H7 decreases with increasing amplitude of the daily temperature oscillations [6]. It has been demonstrated that during temperature cycling *E. coli* membrane composition may become conditioned to cold [10].

The focus of this study is how CspA and CspB respond to a slow temperature fluctuation between the optimal temperature for growth (37°C) and the minimal temperature for growth (8°C) of *E. coli*. Although an educated guess based on the cold shock behaviour and growth at optimal temperature of *E. coli* can be made, the effect of temperature fluctuation on CspA and CspB has not been experimentally verified. The cold shock response in *E. coli* is well documented and provides a good reference point to gauge the extent of the effect of temperature fluctuations on cell physiology [11]. During classical cold shock, CspA protein and its homologous proteins (i.e. CspB, CspG, CspI) are considered to have an essential role in adaptation to low temperatures [11,12]. For instance, CspA and CspB bind to mRNA and work as chaperones to facilitate mRNA translation by preventing formation of mRNA secondary structures [13,14]. CspA also acts as a transcription regulator protein [15,16]. Because CspA and CspB translation does not require cold adapted ribosomes [17], cells respond quickly to cold shock. It was postulated that induction of cold shock genes is primarily due to post-transcriptional regulation [18], while recent investigations have shown that translational preferences during cold shock are present [19-21]. It is also known that *cspA* mRNA and its protein CspA are hardly detected in cells growing at a constant 37°C [22]. In the natural microbial environment, temperature changes are usually gradual. This is different from the classical cold shock experiment where the temperature is rapidly changed and cells do not experience a gradual change of temperature. Slow and fast cold shock may lead to different cell adaptation. There are, however, no systematic studies to show the effect of periodic slowly changing temperature on transcription and translation of CspA and CspB cold shock proteins.

In this work *Escherichia coli* was used as a model system to study proteome and specifically cold shock CspA and CspB protein response to temperature fluctuations. Cells were grown at the optimal growth temperature (37°C) for one hour, then incubated for one hour at the minimal growth temperature (8°C), transferred back to 37°C for one hour and so forth for 12 hours of incubation. The response was compared to cells incubated at a constant 37°C or ones cold shocked at 8°C. The transcription of *cspA* and *cspB* mRNA was determined by real-time PCR. The relative protein concentration and degradation levels of CspA-FLAG and CspB-FLAG were measured by Western and immuno blot analysis. This study represents the first report of the transcription and translation of CspA and CspB during slow periodic temperature fluctuation.

## Methods

### Bacterial strains and plasmids

Proteins CspA and CspB were FLAG tagged as previously described using the plasmids pKD46 and pSUB11 [23] and the C-term FLAG was confirmed by PCR. The pCP20 plasmid was used in order to eliminate the kanamycin cassette in the CspA-FLAG and CspB-FLAG strains [24]. *Escherichia coli* wild type strain K12 MG1655 and *E. coli* K12 ESH10 were used.

### Bacterial growth

Bacteria were grown as described previously [10]. In temperature fluctuation experiments, bacteria were incubated for a total period of 12 hours, with alternating one-hour periods at 37°C and 8°C. In cold shock experiments, bacteria were incubated at 37°C until the mid exponential phase (2.5 h) and then transferred to 8°C for the remaining four hours. In control experiments, bacteria were incubated for 12 hours at a constant 37°C. The temperature in the growth medium oscillated continuously between 37°C and 8°C and was monitored as described previously [10].

### Preparation of bacterial lysates for two-dimensional electrophoresis

*E. coli* K12 ESH10 samples were harvested at different time points and centrifuged (9000 rpm, 10 min, 4°C), washed four times with 10 mM Tris HCl, 250 mM sucrose at pH 7.4. The pellet was resuspended in rehydration buffer (2 M thiourea, 7 M urea, 2% (w/v) CHAPS, 2% (v/v) immobilized pH gradient (IPG) buffer, 65 mM DTT) without bromphenol blue. Cells were lysed using sonication (4 times for 15 s with intermediate ice cooling) and centrifuged (21000 × g, 30 min, 4°C). Concentrations of proteins in the supernatant were determined by the Bradford method (Bradford, 1976). A 2-D Clean Up kit (GE Healthcare) was used for protein extract cleaning before 2-D gel electrophoresis and stored at −80°C until further analysis.

### Two-dimensional gel electrophoresis and gel image analysis

Two-dimensional electrophoresis (2DGE) was performed as described previously [25]. Typically, gels were stained with the fluorescent dye SYPRO Ruby (Invitrogen) and scanned using a G BOX:HR CCD camera (Syngene). Gels were made in triplicate. Gel image analysis was done with 2-D Dymension software version 2.02 (Syngene). The triplicate gels were matched to create an average sample gel. Spots were detected and quantified on the basis of their normalized volume (the spot volume was divided by the total volume over the whole set of gel spots). The average sample gels of temperature treated and control (those incubated at a constant 37°C) cells were compared for differentially produced proteins. The change of production was considered significant only if the intensity of the corresponding spot differed reproducibly by more than two-fold in normalized volume between the treated and control cells and if it was statistically significant (ANOVA test, p < 0.05).

### Protein identification by mass spectrometry

The protein spots that showed significantly differential production were cut from the gels and analysed by a MALDI-TOF/TOF mass spectrometer at the York Technology Facility (University of York, Great Britain). The proteins in the gel pieces were washed twice with 50% (v/v) aqueous acetonitrile containing 25 mM ammonium bicarbonate, then once with acetonitrile and dried in a vacuum concentrator for 20 min. Sequencing-grade, modified porcine trypsin (Promega) was dissolved in the 50 mM acetic acid supplied by the manufacturer, then diluted 5-fold by adding 25 mM ammonium bicarbonate to give a final trypsin concentration of 0.02 g/l. The gel pieces were rehydrated by adding 10 μl of trypsin solution, and after 30 min 25 mM ammonium bicarbonate solution was added to cover the gel pieces, which were incubated overnight at 37°C. A 1 μl aliquot of each peptide mixture was applied directly to the ground steel MALDI target plate, followed immediately by an equal volume of a freshly-prepared 5 mg/ml solution of 4-hydroxy-α-cyano-cinnamic acid (Sigma) in 50% (v:v) aqueous acetonitrile containing 0.1% (v:v) trifluoroacetic acid. Positive-ion MALDI mass spectra were obtained using a Bruker ultraflex III in the reflectron mode, equipped with a Nd:YAG smart beam laser. MS spectra were acquired over a mass range of m/z 800–4000. The final mass spectra were externally calibrated against an adjacent spot containing 6 peptides (des-Arg^1^-Bradykinin, 904.681; Angiotensin I, 1296.685; Glu^1^-Fibrinopeptide B, 1750.677; ACTH (1–17 clip), 2093.086; ACTH (18–39 clip), 2465.198; ACTH (7–38 clip), 3657.929.). Monoisotopic masses were obtained using a SNAP algorithm (C 4.9384, N 1.3577, O 1.4773, S 0.0417, H 7.7583) and a S/N threshold of 2. For each spot the ten strongest peaks of interest, with an S/N ratio greater than 30, were selected for MS/MS fragmentation. Fragmentation was performed in the LIFT mode without the introduction of a collision gas. The default calibration was used for MS/MS spectra, which were baseline-subtracted and smoothed (Savitsky-Golay, width 0.15 m/z, cycles 4); monoisotopic peak detection used a SNAP averaging algorithm (C 4.9384, N 1.3577, O 1.4773, S 0.0417, H 7.7583) with a minimum S/N of 6. Bruker flexAnalysis software (version 3.0) was used to perform the spectral processing and peak list generation for both the MS and MS/MS spectra. The tandem mass spectral data were submitted to database searching using a locally-running copy of the Mascot program (version 2.1; Matrix Science), through the Bruker BioTools interface (version 3.2). Criteria to search the NCBInr database (version 2008. 09. 24; 7044224 sequences, 2433775060 residues) included tryptic digest with a maximum number of one missed cleavage and monoisotopic peptide masses. The mass tolerance was set to ± 100 ppm after internal calibration and the fragment mass tolerance to ± 0.4 Da. Additionally, carbamidomethylation was set to fixed modification and oxidation of methionine, and was considered as a variable modification. Peptide identifications with an expected score less than 0.05 were accepted. Protein homology identifications for the top hit (first rank) that contained at least one peptide with an ion score greater than the MOWSE identity threshold at p < 0.05 were retained. For protein matches with only one unique peptide, the peptide sequence was searched against the non-redundant protein database in the National Center for Biotechnology Information (NCBI) BLASTp (taxonomy was limited to *Escherichia coli*) to ensure that no other proteins share exactly the same peptide sequence.

### RNA isolation

Total RNA from *E. coli* K12 MG1655 was isolated using the "RNA Protect" and "RNeasy" kits (Qiagen) according to the manufacturer's instructions. DNase I (Qiagen) was used to remove genomic DNA contamination.

### Quantitative real-time PCR

One microgram of the DNase-treated total RNA was reverse transcribed using random hexamers (Fermentas) by ImPromII reverse transcriptase (Promega). To quantify *cspA* transcript levels the forward primer 5'- GGCTTCGGCTTCATCACTCCTG -3' and the reverse primer 5'- TACCAGCTGCCGGGCCTTTAG -3' were constructed. PCR reactions were performed using 250 nM of each gene-specific primer in a 20 μl volume with 1× SYBR green PCR master mix (Thermo Scientific). The reactions were run on a Corbett Research instrument using the following cycling parameters: 95°C for 15 min, 40 cycles of denaturation at 95°C for 30 s, primers annealing at 56°C for 20 s and extension at 67°C for 25 s. To measure the *cspB* transcript levels, the forward primer 5'- TGCATTTTTCTGCGATTCAG -3' and the reverse primer 5'-GCAGGACCTTTAGCACCACT-3' were used. PCR reactions were performed using 250 nM of each gene-specific primer in a 20 μl volume with 1× Power Sybr green PCR master mix (Applied Biosystems). Reactions were run on an ABI prism 7900 HD (Applied Biosystems) as described by the manufacturer. The relative amounts of *cspA* and *cspB* transcripts were calculated by the ΔC_q_ method [26].

### Degradation assay

Spectinomycin (300 μg/ml) was added to *E. coli* K12 MG1655 at different time points (*t =* 5 h and 6 h during temperature fluctuations, *t =* 2.5 h at 37°C, and 1 h after temperature downshift from 37°C to 8°C in cold shock experiments). The protein samples were taken every 30 minutes after the addition of antibiotic. Aliquots containing equal amounts of protein were fractionated by sodium dodecyl sulphate-polyacrylamide gel electrophoresis (SDS-PAGE) and detected by the Western and immuno blot analysis as further described.

### Western and immuno blot analysis

Following SDS-PAGE using 12% polyacrylamide gel, proteins were transferred onto a nitrocellulose membrane (Whatman) using the wet method. Mouse anti-FLAG (Sigma) was used as the primary antibody (1:1000) and horseradish peroxidase-conjugated goat anti-mouse IgG antibodies (Jackson ImmunoResearch) as secondary antibodies (1:10000). An enhanced chemiluminescence detection kit (Pierce) was used for protein detection on the membranes. The samples were scanned by a G BOX:HR CCD camera (Syngene) and analysed with GeneTools (Syngene). A calibration curve for CspA-FLAG protein was prepared. There was a linear increase of signal in the total cellular protein range from 0 to 240 μg (R^2^ = 0.92). At 60 μg of total cellular protein used for all the samples in this work, the Western assay was shown to be within the linear dynamic range.

## Results

**Figure 1:**
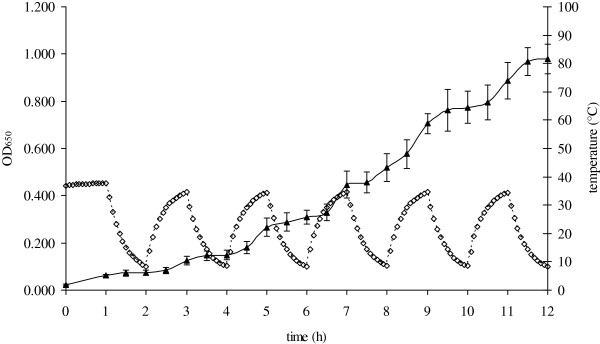
**Growth of bacteria *E. coli *and temperature fluctuation during periodic temperature cycling.** Triangles symbolize bacterial growth in LB medium, diamonds represents temperature measured during periodic temperature cycling between 37°C and 8°C with a period of 2 h.

In one set of experiments the temperature in the growth medium of *E. coli* continuously oscillated between 37 and 8°C (Figure 1), resulting in a step-like growth curve. Bacterial growth decreased during each temperature downshift, when the temperature of the bacterial growth medium dropped to 15°C or lower. Similarly during temperature upshift, growth resumed after the temperature in the growth medium reached 20°C or higher.

**Figure 2:**
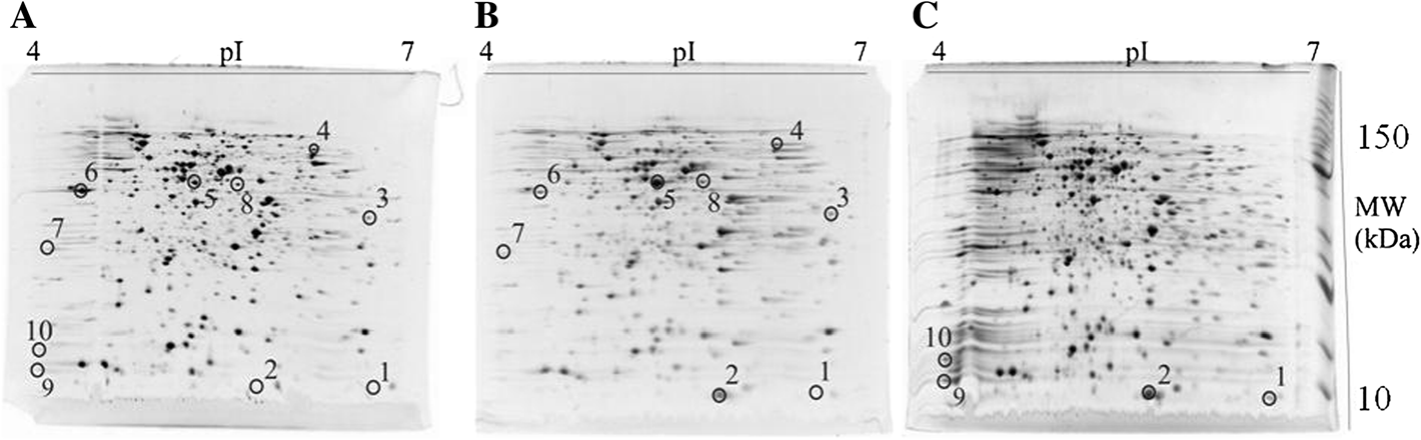
**2DGE gel of total cell protein from *E. coli *during periodic temperature cycling.** Two dimensional gels of *E.coli* lysates obtained during constant incubation at 37°C **(A)**, periodic temperature cycling **(B)**, and at 8°C **(C)**. The pI range is 4–7 and the molecular mass range is 10–250 kDa. The identity of protein spots that were significantly (p>0.05) up or down produced relative to the proteins in cells constantly incubated at 37°C are given in Table 1.

The effect of the periodic temperature cycling on cellular protein profile was studied by two-dimensional electrophoresis (2DGE). The protein profile after five hours of temperature cycling (Figure 2) was compared to the protein profile of cells incubated at a constant 37 or 8°C. Based on this 2-D analysis, the intensities of several spots in the temperature cycled cells were changed, but only proteins which had 2-fold or greater increased or decreased production on temperature cycling were selected for further identification and study. The protein identity determined by mass spectrometry and their assigned physiological functions are given in Table 1. The proteins that were significantly (p < 0.05) up or down regulated during thermo-cycling belong to cold stress response proteins, proteins involved in energy metabolism, transport proteins, and proteins involved in amino acid synthesis. The response of the cold shock proteins CspA and CspB was further characterized.

**Table 1:** Protein identification

**General function**	**Spot number**	**Protein name or function**	**NCBInr accession no.**	**Gene**	**Up or down regulation**		**MOWSE score**	**Sequence coverage (%)**
					**temperature cycled cells relative to cells constantly at 37°C**	**cold shock cells relative to cells constantly at 37°C**		
Stress response	1	Cold shock-like protein cspB	gi|16129516	*cspB*	*de novo*	*de novo*	301	52
	2	Cold shock protein cspA	gi|15804102	*cspA*	+ 5.1	+ 2.8	310	52
Energy metabolism	3	Succinyl-CoA synthetase subunit alpha	gi|1580008	*sucD*	+ 2.7	NC	105	5
Peptide transport	4	Periplasmic oligopeptide-binding protein	gi|1583099	*oppA*	+ 3.1	NC	340	11
Sugar transport	5	Maltose-binding periplasmic protein	gi|15834271	*malE*	+ 5.8	NC	519	17
Ion transport	6	Outer membrane porin protein nmpC	gi|145856	*nmpC*	- 10.0	NC	555	26
Electron transport	7	Flavodoxin-1	gi|2781032	*fldA*	- 2.1	NC	532	38
Amino acid biosynthesis	8	Phosphoserine aminotransferase	gi|3891552	*serC*	- 2.4	NC	260	14
Protein synthesis	9	30S ribosomal protein S16	gi|15803131	*rpsP*	NC	+ 3.6	96	19
	10	50S ribosomal protein L27	gi|15833318	*rpmA*	NC	+ 2.5	112	16

Proteins that were significantly (p < 0.05) up (+) or down (−) regulated during the periodic temperature cycling or the cold shock relative to the protein levels incubated constantly at 37°C. NC (not changed).

**Figure 3:**
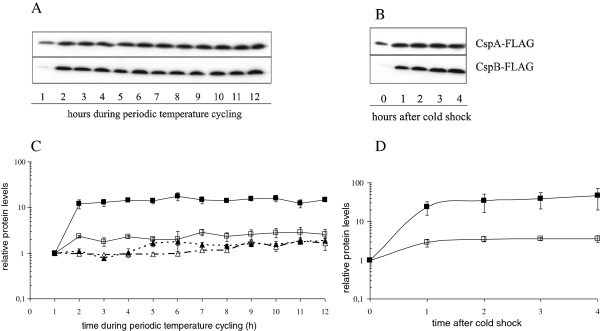
**Western blot assayof CspA-FLAG, CspB-FLAG and the graphical presentation.** Western blot assay during the periodic temperature cycling **(A)** and during the cold shock **(B)** is shown. The relative amounts of CspA-FLAG (empty squares), CspB-FLAG (full squares), the relative total protein concentration in CspA-Flag cells (empty triangles), and the relative total protein concentration in CspB-FLAG cells (crosses) during the periodic temperature cycling **(C)** and during the cold shock **(D)** are shown. The amounts of proteins are plotted relative to the level prior to the first temperature down shift, whereas protein values during the cold shock are normalized to the protein values just before temperature downshift. The data were obtained from three independent experiments. Mean values and standard deviation are given.

The relative production of FLAG-tagged CspA and CspB proteins during thermo-cycling was monitored by the Western blotting assay (Figure 3A and C). The relative production of CspA-FLAG and CspB-FLAG proteins increased during the first temperature downshift. Subsequent temperature oscillations did not significantly (p > 0.05) change the relative protein levels. The relative response of CspB-FLAG was approximately 10-fold higher than of CspA-FLAG. On the other hand, the normalized total protein concentration increased toward the end of the incubation experiment as expected. The results therefore indicate that the ratio of CspA or CspB to total protein was not constant but changed during the incubation. It first significantly increased and then slowly decreased toward the end of incubation, but never reached the initial ratio.

**Figure 4:**
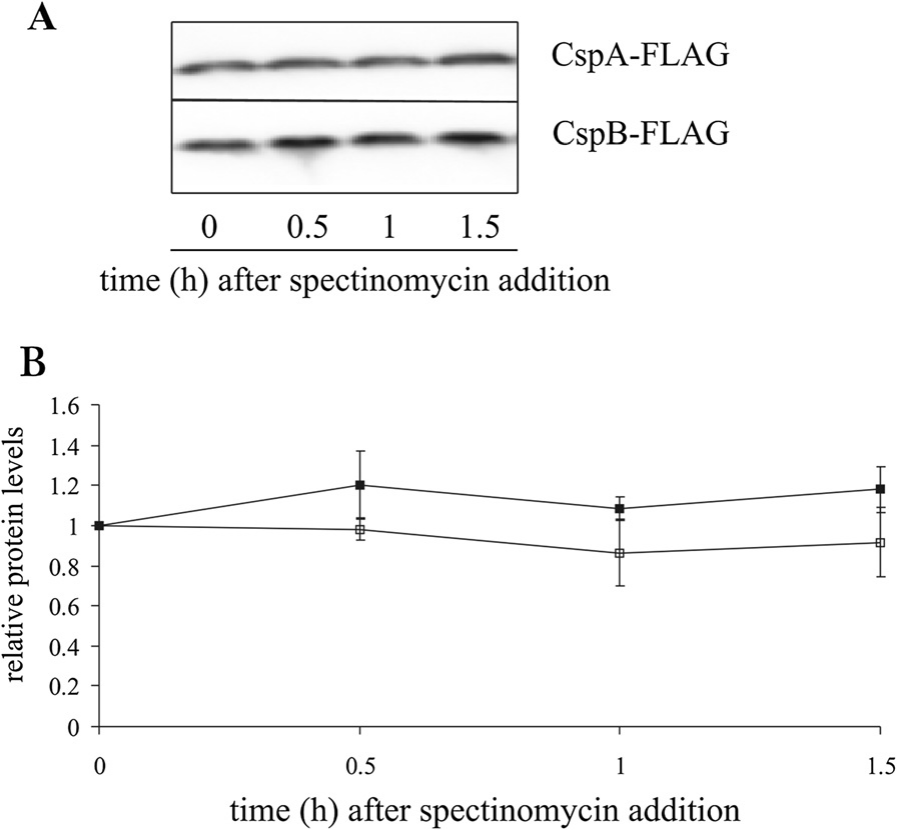
**The degradation of CspA-FLAG and CspB-FLAG.** Western blot assay after five hours of incubation when cells had completed three temperature cycles **(A)**, and a graphical representation of the degradation of CspA-FLAG (empty squares) and CspB-FLAG (full squares) are shown **(B)**. The amounts of proteins are plotted relative to the amount prior to the addition of spectinomycin. The data were obtained from three independent experiments. Mean values and standard deviations are given.

The corresponding protein production in cells exposed to cold shock is given in Figures 3B and D. As expected cold shock increased the production of both CspA-FLAG and CspB-FLAG proteins. Their relative response was similar to the temperature cycled cells. The degradation of CspA-FLAG and CspB-FLAG proteins was measured after protein synthesis in the cell was blocked by the addition of the antibiotic spectinomycin. As shown in Figure 4, there was no significant protein degradation of CspA- or CspB-FLAG after five hours of incubation. In addition, the CspA-FLAG decay rate was measured at different times and temperatures during the experiment. Samples were taken after 5 h or 6 h during the temperature fluctuation experiment (i.e. at 37 and 8°C), or from cells constantly incubated at 8 or 37°C. Despite their different history, the proteins did not decay when tested by the degradation assay.

**Figure 5:**
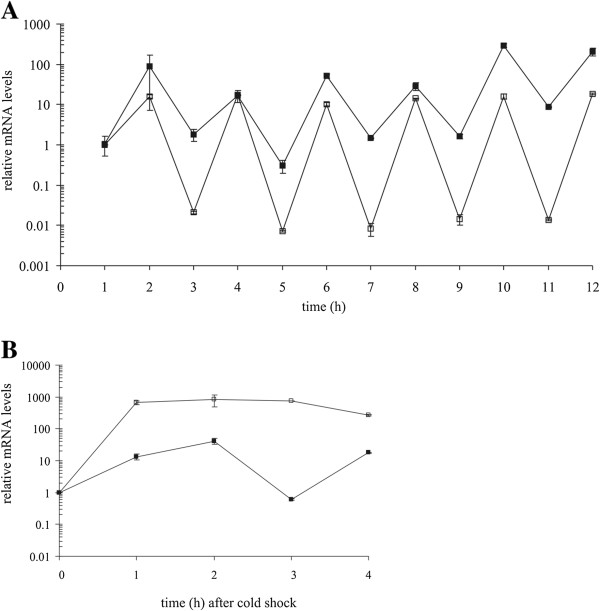
**Gene expression of *cspA *and *cspB.*** The relative expression of *cspA* (empty squares) and *cspB* mRNA (full squares) during the periodic temperature cycling **(A)** and during the cold shock **(B)** is shown. The data are normalized relative to transcript levels prior to the first temperature down shift. The mean values and standard deviations are given.

The relative amounts of *cspA* and *cspB* mRNA transcripts during the periodic temperature cycling and during cold shock are given in Figure 5. In sharp contrast to the relative protein production, the relative transcript levels fluctuated in synchrony with the oscillating temperature. The levels of *cspA* and *cspB* mRNA transcripts increased significantly (p < 0.05) after each temperature downshift. On the other hand, the levels of both transcripts significantly (p < 0.05) decreased during each temperature up-shift. The fluctuation amplitude of the *cspA* mRNA transcript was approximately constant throughout the experiment. An approximately three orders of magnitude difference in *cspA* mRNA transcript levels was observed between low and high temperatures. The corresponding change for *cspB* mRNA was two orders of magnitude. The level of *cspA* transcript during the cold shock experiment significantly increased after temperature downshift and remained approximately at the same level for the next 4 hours. On the contrary, the level of *cspB* transcript first increased and then decreased after 3 hours to the level before the cold shock. Interestingly, after 4 hours at low temperatures the *cspB* transcript level increased again.

## Discussion

In this work temperature was periodically fluctuated between 37°C and 8°C during the growth of *E. coli*, generating continuous physiological stress on the bacterial cells*.* The two temperatures represent the optimal temperature for growth (37°C) and the minimal temperature (8°C) that sustains growth of *E. coli*[27]. During one temperature cycle cells spent approximately one hour at temperatures known to induce cold shock response in *E. coli*[28], when transient arrest of bacterial growth is expected (Figure 1) [29,30].

The results indicate that several proteins were up or down regulated during the thermo-cycling (see Figure 2, Table 1) in comparison to cells incubated at a constant 37°C in the middle of the exponential phase. The cold shock protein CspA was up regulated, while CspB was *de novo* synthesized. This could be the result of different thermoregulation of the two proteins [31]. In addition there was a significant up regulation of succinyl-CoA synthetase subunit alpha (SCS-α), which is a key metabolic enzyme in the tricarboxylic cycle (TCA), and significant down regulation of the electron transport flavodoxin-1 protein. This suggests that the cells were energy stressed [32,33] during temperature cycling. During the thermo-cycling proteins that are involved in transport pathways such as periplasmic oligopeptide-binding protein (OppA) or maltose-binding periplasmic protein (MalE) were significantly up regulated, while the outer membrane porine protein (NmpC) was down regulated. It has been reported [33] that during temperature downshift the proteins involved in amino acid biosynthesis were induced. This might be the result of repressed growth at low temperature and cell effort to overcome metabolic imbalance. Our results, however, showed significant down regulation of phosphoserine aminotransferase (SerC), but only during the periodic temperature cycling. There was no corresponding down regulation during cold shock. At low temperature many different 30S and 50S ribosomal proteins are known to be inhibited [32,33]. In addition, the proteins involved in protein folding are induced during cold shock that prepares the cells to reinitiate protein synthesis during exposure to cold temperatures [34]. Consistently, 30S ribosomal protein S16 and 50S ribosomal protein L27 were up regulated during cold shock. But interestingly, no up regulation of ribosomal proteins was found during the periodic temperature cycling. As has been shown by Jones 1989, approximately two dozen new proteins were produced during classical cold shock [28]. As described in the present work several new proteins were produced during the thermo-cycling but only CspA overlapped significantly with the known cold shock proteins [35]. Since we adopted a rather stringent criterion to label a protein as a cold shock protein (the protein concentration had to increase at least two-fold), it is possible that other cold shock proteins were produced to a lesser degree and more overlap exists between classical cold shock and the cold shock described in this work. If we combine the results it is clear that temperature fluctuation has a profound effect on protein synthesis that could have not been predicted from the results in cells grown at a constant 37°C or during cold shock.

The mechanism of regulation of *cspA* and *cspB* transcription during periodic temperature cycling is not known. In this study the transcript levels of *cspA* and *cspB* mRNA were high at low temperature and dramatically decreased at high temperature during each temperature fluctuation period. This is not very surprising since *cspA* mRNA is constitutively transcribed [36], but as Goldenberg et al. [37] reported, *cspA* mRNA is rapidly degraded at 37°C (a half-life of approximately 10 s) and is not translated. On the other hand, at low temperatures *cspA* mRNA becomes much more stable (a half-life > 20 min). It is generally accepted that post-transcriptional regulation of cold shock genes has a key role in cold shock response [18,19]. It was also shown that the increased *cspA* mRNA stability during cold shock is the result of the functionally distinct mRNA secondary structure at low temperature, which can act as a thermosensor [38]. Therefore the results indicate that the same mechanism of RNA remodelling is also present during temperature cycling.

On the other hand, the major result of this work is the observation that CspA-FLAG and CspB-FLAG proteins did not follow the on-off regulation of *cspA* and *cspB* during temperature fluctuations. The results indicate that CspA-FLAG and CspB-FLAG levels in thermo-cycled cells were higher compared to cells incubated at a constant 37°C. It is generally accepted that CspA and CspB proteins are abundantly produced at low temperatures but only sparsely at high temperatures [31,39,40]. The results of the periodic temperature cycling experiments are therefore unexpected and could not be predicted in advance. It is possible that CspA is a rather stable protein that is not degraded at high temperatures and can accumulate in the cell (Figure 3). Since CspA is mainly produced at low temperatures, it is interesting to note that apart from the first temperature down shift its level remains unchanged during temperature cycling. The absence of a relative increase in CspA levels during thermo cycling may be the result of reduced translation of the cold shock proteins [19]. However, if no cold shock protein synthesis were to occur during temperature cycling, both CspA and CspB proteins would be diluted in the progeny cells. Since this has not been observed, we speculate that some protein synthesis must have occurred. As shown in Figure 1, cells resume growth after each temperature upshift. There are two possible mechanisms that could increase CspA protein synthesis in the cell. Apart from cold shock, CspA is also synthesized on carbon-source upshift at 37°C, which increases the growth rate of *E.coli*. The increased transcription of *cspA* is a result of the close proximity of *cspA* to the origin of replication *oriC*[41,42]. This in turn leads to a higher gene dosage effect and higher *cspA* mRNA stability due to lower RNase activity, and allows CspA to reach one-sixth of its cold-shocked induction level [39,41]. Since growth occurred after each temperature upshift when the temperature in the growth medium reached 20°C or higher, it is possible that some CspA could be synthesized due to the higher gene dosage effect after growth resumed.

Although much less is known about the behaviour of the CspB protein, the results of this study indicate that at least qualitatively it behaves similarly to CspA protein. The stronger response of CspB relative to CspA might be the result of different thermoregulation, since it is known that CspA is more induced than CspB at temperatures above 30°C [31]. It is, however, less likely that the same mechanism of induction would be in operation for CspB during temperature cycling as for CspA, because CspB is not located near the *oriC*[42].

## Conclusions

Protein production during temperature cycling is different from protein production either at 37°C or during cold shock. The results indicate consistently higher levels of *cspA* and *cspB* transcripts after each temperature downshift and up to three-orders of magnitude lower levels after each temperature upshift due to decreased mRNA stability at high temperatures. There was no corresponding fluctuation of CspA and CspB protein concentrations during the temperature cycling. It was observed that proteins were not degraded during temperature up shift. Although microbiologists have learned a great deal from experiments conducted at constant temperatures, the results of this study show that bacterial physiological response to fluctuating temperatures is not a simple superimposition of the results obtained at different temperature conditions.

## Competing interests

The authors declare that they have no competing interests.

## Authors’ contributions

TI carried out the experiment, PJ participated in the 2DGE and helped with protein identification. DS participated in the design of the study. TI, PJ and DS wrote the manuscript. All authors read and approved the final manuscript.
